# The role of peptides in reversing chemoresistance of breast cancer: current facts and future prospects

**DOI:** 10.3389/fphar.2023.1188477

**Published:** 2023-05-22

**Authors:** Yongxiu Huang, Hongyao Peng, Anqi Zeng, Linjiang Song

**Affiliations:** ^1^ School of Medical and Life Sciences, Chengdu University of Traditional Chinese Medicine, Chengdu, China; ^2^ Institute of Translational Pharmacology and Clinical Application, Sichuan Academy of Chinese Medical Science, Chengdu, Sichuan, China

**Keywords:** breast cancer, drug resistance, peptide, apoptosis, chemotherapy

## Abstract

Breast cancer is the first malignant tumor in women, and its incidence is also increasing year by year. Chemotherapy is one of the standard therapies for breast cancer, but the resistance of breast cancer cells to chemotherapy drugs is a huge challenge for the effective treatment of breast cancer. At present, in the study of reversing the drug resistance of solid tumors such as breast cancer, peptides have the advantages of high selectivity, high tissue penetration, and good biocompatibility. Some of the peptides that have been studied can overcome the resistance of tumor cells to chemotherapeutic drugs in the experiment, and effectively control the growth and metastasis of breast cancer cells. Here, we describe the mechanism of different peptides in reversing breast cancer resistance, including promoting cancer cell apoptosis; promoting non-apoptotic regulatory cell death of cancer cells; inhibiting the DNA repair mechanism of cancer cells; improving the tumor microenvironment; inhibiting drug efflux mechanism; and enhancing drug uptake. This review focuses on the different mechanisms of peptides in reversing breast cancer drug resistance, and these peptides are also expected to create clinical breakthroughs in promoting the therapeutic effect of chemotherapy drugs in breast cancer patients and improving the survival rate of patients.

## 1 Introduction

Breast cancer is one of the global public health problems (Oo et al., 2021), and it is also the most common cancer among women in the world (Wang et al., 2017). By October 2022, breast cancer had surpassed lung cancer to become the most common malignant tumor in the world. Its occurrence is related to many factors, involving a variety of genetic and epigenetic changes (Li et al., 2022). Breast cancer can be divided into different subtypes. From the purpose of treatment, breast cancer can be divided into three subtypes: human epidermal growth factor receptor 2 (HER2) positive, androgen and progesterone receptor (ER, PR) positive, and triple-negative (Weigelt and Reis-Filho, 2009). Triple-negative breast cancer (TNBC), also known as triple-negative breast cancer (TNBC), is named for the absence of estrogen receptor (ER), progesterone receptor (PR), and human epidermal growth factor receptor 2 (HER2) (Yeo and Guan, 2017). Breast cancer treatment methods include surgical resection, chemotherapy, radiotherapy, endocrine therapy, and targeted therapy, and chemotherapy is one of the standard therapy (Wang et al., 2017), especially nowadays breast-conserving surgery is performed with neoadjuvant chemotherapy without resection (Li et al., 2014). Cytotoxic drugs are often used as first-line drugs, such as doxorubicin (DOX), methotrexate (MTX), cisplatin (CDDP), paclitaxel (PTX), etoposide, and so on (Wang et al., 2017). The mechanisms of action of these chemotherapeutic drugs are different. Doxorubicin is an anthracycline drug, and its mechanism of action mainly involves the insertion of chromosome and mitochondrial DNA and the inhibition of topoisomerase II. Methotrexate is a folate antagonist that inhibits dihydrofolate reductase, so the lack of reduced folate substrate impairs the synthesis of purine nucleotides, thereby inhibiting DNA synthesis and ultimately reducing cancer cell proliferation (Lindgren et al., 2006). Paclitaxel is a common microtubule inhibitor, mainly by destroying the mitotic spindle to activate the mitotic checkpoint to prevent cell mitosis, resulting in cell stagnation during mitosis (Singh et al., 2007). These chemotherapy drugs appear early, cancer cells are particularly sensitive to them, effectively inhibit tumor growth and metastasis, and prolong the life of patients in clinically achieved good results. However, with the widespread use of chemotherapy drugs, the joy brought by good therapeutic effects gradually disappeared (Vasan et al., 2019), followed by cancer recurrence, tumor cell metastasis, and uncontrollable conditions. This is the resistance of tumor cells to chemotherapeutic drugs.

Because of the resistance to chemotherapeutic drugs, although the treatment strategy of combined use of multiple chemotherapeutic drugs was later adopted, the effect was still poor (Vasan et al., 2019). Drug resistance can be divided into intrinsic resistance, acquired resistance, and multidrug resistance, and multidrug resistance was later considered to be the most common. Multidrug resistance often leads to poor treatment and poor prognosis of these chemotherapeutic drugs for solid tumors such as breast cancer through various molecular mechanisms (Haggag et al., 2020). According to the study, the response rate of metastatic breast cancer to first-line chemotherapy drugs is usually 30%–70%, but it is not persistent. Generally, drug resistance occurs in 6–10 months, resulting in treatment failure. The 5-year survival rate of patients with metastatic breast cancer is only 27% (Wang H. et al., 2014). Therefore, exploring the molecular mechanism of multidrug resistance of chemotherapeutic drugs has also become a research hotspot. At present, several common drug resistance mechanisms have been studied, including the disorder of the apoptosis mechanism, the activation of the DNA repair mechanism, the mutation of the drug target, and the regulation and disorder of drug inflow (Haggag et al., 2020). For example, cancer cells transport P53 to the cytoplasm through the CRM1-mediated output mechanism to escape the apoptotic effect of P53 (Haggag et al., 2020), resulting in decreased cytotoxicity of drugs such as doxorubicin. For another example, proliferating cell nuclear antigen is involved in DNA replication, DNA repair, DNA methylation, chromatin remodeling, cell cycle regulation, and protein degradation (Lingeman et al., 2014), making cisplatin-induced cell death ineffective. And the effectiveness of drugs like paclitaxel is limited by resistance mechanisms partly mediated by the upregulation of anti-apoptotic BCL-2 and P-glycoprotein (Gupta et al., 2018). In addition, the current chemotherapy is non-specific, regardless of the subtypes of breast cancer, but in terms of drug resistance, for specific types of TNBC, the mechanism of resistance to chemotherapy drugs due to heterogeneity is very complicated (Bakrania et al., 2016). Therefore, the current research is more in the exploration of specific targeted signal transduction pathway therapy, such as a specific *β*-interferon inducer DEAE-glucan can be targeted to deliver doxorubicin to tumor tissue (Bakrania et al., 2018).

In recent studies, peptides, especially bioactive peptides, are a hot spot in reversing the resistance of breast cancer cells to chemotherapeutic drugs. Peptide is an amino acid with a molecular weight between protein and small molecule (Zhang et al., 2023). It is generally a short chain with more than 50 amino acids, usually containing a disulfide bond. Its sequence and structure are adjustable to increase its interaction with specific molecules. Peptides related to cancer treatment can be divided into three groups: cell-penetrating peptide (CPP), tumor-targeting peptide, and pore-forming peptide (Beheshtirouy et al., 2021). Bioactive peptides are peptides that can bind to molecular targets and affect cells or organisms. They have many favorable properties such as high tissue penetration, good biocompatibility, and good binding affinity with target molecules (Oo et al., 2021). Of course, this has to mention the bioactive cationic peptide (BCP). BCP is widely distributed in nature. The positively charged residues on BCP can interact with the negatively charged groups on the tumor membrane, and then the acyl chain of the lipid membrane. The hydrophobic interaction between non-polar residues induces the instability of the lipid bilayer and structural and physicochemical changes, which in turn leads to cell death (Manrique-Moreno et al., 2021). There are also bioactive peptides from natural aquatic products, including marine peptides, which have therapeutic potential in breast cancer (Ahmed K. S. et al., 2021). Given the different mechanisms of drug resistance and the different roles played by peptides, we will elaborate on the mechanism of different peptides reversing the resistance of breast cancer to first-line chemotherapy drugs in the six aspects of promoting cancer cell apoptosis, promoting non-apoptotic regulatory cell death of tumor cells, hindering DNA damage repair, affecting tumor microenvironment, inhibiting the efflux mechanism of tumor cell chemotherapy drugs and increasing the uptake of chemotherapy drugs Table 1. To study the reversal of drug resistance of breast cancer by peptides, improve the therapeutic effect of chemotherapy and control the metastasis of tumor cells. Scholars who improve the survival rate of breast cancer patients provide references.

**TABLE 1:** Mechanism of peptide reversing chemotherapy resistance of breast cancer.

Catalog		Peptide name	Source	Peptide sequence	Reversed drugs	Reversing resistance mechanisms	References
Apoptosis pathways	Apoptosis gene	anti-HSP70 peptide	chemical synthesis	YCAYYSPRHKTTF	DOX	Heat shock protein 70 (HSP70)	Tan et al. (2021)
		NuBCP-9 peptide	Nur77	_	DOX	Exposing the BCL-2 BH3 domain	Gupta et al. (2018)
		AVPIR8	chemical synthesis	FMOC-Arg (pbf)-OH, FMOC-Ala-OH, FMOC-Val-OH, FMOC-Pro-OH, FMOC-Ile-OH	DOX	p53 ↑, a high binding affinity to the X-linked IAPs	Wang et al. (2014a)
	Soluble membrane peptide	Membrane-Lytic Peptide	chemical synthesis	GLLxLLxLLLxAAGW	DOX	ACPs, anionic residues	Chen et al. (2022b)
		HNPs-1	neutrophils, specific subsets of T cells, monocytes, and NK cells	_	DOX	↑ plasma membrane permeability	Li et al. (2014)
		Heterochiral β-Peptide Polymers	chemical synthesis	_	DOX	Destroying the cell membrane	Bakrania et al. (2017)
	Mitochondrial apoptosis pathway	HNPs-1	neutrophils, specific subsets of T cells, monocytes, and NK cells	_	DOX	Destruction of mitochondrial transmembrane potential (△ Ψm)	Li et al. (2014)
		HER-2 peptide	chemical synthesis	YCDGFYACYMDV	DOX	The generation of ROS and damage to mitochondrial respiratory chain components	Shi et al. (2018)
		R 8 H 3 peptide	chemical synthesis	MLRAALSTARRGPRLSRLLHHHRRRRRRRR	DOX	Achieved effective reactive oxygen species (ROS)	Ahmed et al. (2021a)
	Nuclear transcription factor	The peptide aptamer AII-7	chemical synthesis	LNFYRHGFLPNAVMASMLEVGPWFELLGLCGLAGHPLSSLRI	PTX	Interference with AKT activation; ↓ FOXO-1 transcription factor, nuclear factor nB	Kunz et al. (2006)
		T10-ERK	chemical synthesis	HAIYPRHGGCGMPKKKPTPIQLNP	DOX	↓ extracellular signal-regulated kinases (ERK)	Sheng et al. (2016)
		EN1-iPep	the EN1 transcription factor	N-KKKRKVPLVWPAWVYCTRYSDR-C	DTX	↓ homeobox transcription factor Engrailed 1 (EN1)	Sorolla et al. (2016)
	Cell cycle arrest	ANK peptide	chemical synthesis	KGNSALHVASQHGHLGCIQTLVRYGANVTMQNHG	PTX	SNCG with BubR1 interaction	Singh et al. (2007)
		NT21MP	chemical synthesis	H-D-leu-D-Gly-D-Ala-D-Ser-D-Trp-Dhis-D-Arg-D-Pro-D-Asp-D-Lys-Cys-Cys-Leu-Gly-Tyr GlnLys-Arg-Pro-Leu-Pro-OH	PTX	miR-335 ↑; SETD8 ↑, Wnt/β-catenin signaling, genes cyclin D1 and G0/G1	Wang et al. (2017)
		ALT	ALT cell lines	_	palbociclib	↓ the tyrosine phosphorylation of p27Kip1(CDKN1B), CDK4, CDK2, G1	Jilishitz et al. (2021)
Non-apoptotic regulatory cell death		ALT	ALT cell lines	_	palbociclib	↑ RIPK1 phosphorylation; ↓ Ca2+ ion channels and Na + ion channels	Jilishitz et al. (2021)
		Acid-Sensitive Peptide	The sequence of cell surface proteins	HAIYPRHGGC, THRPPMWSPVWPGGC	DOX	cathepsin D ↑; caspase-3 ↑	Sheng et al. (2015)
DNA repairing mechanism		caPeptide	proliferating cell nuclear antigen (PCNA)	RRRRRRRRRCCLGIPEQEY, RRRRRRRRRCCEPGLIYEQ	CDDP	↓ proliferating cell nuclear antigen (PCNA)	Lingeman et al. (2014)
		RanGTP inhibitory peptide (RAN-IP)	Ran protein sequence	CAQPEGQVQFK	DOX	↓ Ras-related nuclear protein (Ran-GTP)	Haggag et al. (2020)
Tumor microenvironment		anti-PN peptides	chemical synthesis	TFATHGKHWAAP	DOX	↑ phosphorylation of AKT, ↑ expression of survivin	Oo et al. (2021)
		Abstract. Human neutrophil peptides-1 (HNPs-1)	chemical synthesis	_	DOX	↓ vivo tumor angiogenesis	Li et al. (2014)
		D-CAN	bariatric surgery patients	CSWKYWFGEC	CDDP, PTX	↓ Cancer-associated fibroblasts (CAFs); Extracellular matrix (ECM) remodeling, vascularization; Immunosuppression	Su et al. (2020)
Efflux mechanism		T10-ERK	chemical synthesis	HAIYPRHGGCGMPKKKPTPIQLNP	DOX	↓ ATP-binding cassette (ABC) transporters such as P-glycoprotein (P-gp)	Sheng et al. (2016)
		TAT	chemical synthesis	CGGGYGRKKRRQRRR	DOX	bypassing the efflux pro- tein (e.g., P-glycoprotein)	Mozaffari et al. (2021)
		NuBCP-9	Nur77	_	PTX	Decomposition of Pgp1 receptor	Gupta et al. (2018)
Enhanced drug uptake	Tumor imaging and drug-delivery	CendR peptide	chemical synthesis	Cys-Arg-Gly-Asp-Lys	CDDP	Binding to the neuropilin-1 receptors (Nrp-1)	Bai et al. (2019)
		Novel peptide	chemical synthesis	NH2-WxEYAAQkFL-CONH2	DOX	Two D-amino acid substitutions at the enzymatic labile sites	Soudy et al. (2013)
		Targeting Peptide	chemical synthesis	Gly−Phe−Leu−Gly, GFLG	DOX	Cathepsin B ↑	Zhi et al. (2019)
		Cancer-selective tetra-branched peptides	chemical synthesis	pyroELYENKPRRPYIL-NH	MTX	Binding to the receptor-related proteins (LRP) receptors and heparan sulfate chains on membrane proteoglycans	Depau et al. (2017)
		bombesin peptide Bombesin (Bn)	chemical synthesis	_	DOX	Binding to the GRPR receptors	Wang et al. (2016)
		T10-EKP	chemical synthesis	HAIYPRHGGCGMPKKKPTPIQLNP	DOX	Binding to the transferrin receptor (TfR)	Sheng et al. (2016)
		YTA2 and YTA4	chemical synthesis	Acetyl-YTAIAWVKAFIRKLRK-amide Acetyl-IAWVKAFIRKLRKGPLG-amide	MTX	Cell-penetrating peptides CPP, across the plasma membrane	Lindgren et al. (2006)
		HER-2 peptide	chemical synthesis	YCDGFYACYMDV	DOX	Binding to the human epidermal growth factor receptor-2 (HER-2)	Shi et al. (2018)
		Novel TIMP3 Peptide p700	tissue inhibitor of metalloproteinase 3 (TIMP3)	Azido-PEG12-KIKSCYYLPCFVTSKN-Lys (5-FITC)-amide	DOX	Binding to the VEGFR1	Aldughaim et al. (2020)
		Nap-GFFpYK peptide	chemical synthesis	_	etoposide	↑ solubility, phosphoric acid group	Zhang et al. (2020)
	Only as a carrier	cyclic peptides (CPs)	chemical synthesis	-L-Gln-D-Ala-L-Glu-D-Ala-LGln-D-Ala-L-Cys-D-Ala-	DOX	High drug encapsulation ratio	Wang et al. (2014b)
		amphiphilic peptide dendrimers (AmPDs)	chemical synthesis	_	DOX	Efficient encapsulation	Zhu et al. (2021)

## 2 Peptides reverse drug resistance: promote apoptosis in breast cancer cells

The imbalance of apoptosis is the main reason for the accumulation of immortalized cells and the promotion of cancer development. Of course, it is also related to chemotherapy resistance (Cotter, 2009). Therefore Promoting apoptosis of cancer cells is a good way to improve the sensitivity of tumors to chemotherapeutic drugs (Wang H. et al., 2014), and there are several ways to induce apoptosis. Here, five main apoptotic pathways are mentioned, namely: regulation of apoptotic genes, lysosomal peptide apoptotic pathway, mitochondrial apoptotic pathway, nuclear transcription factor-involved apoptosis, and cell cycle arrest apoptotic pathway. Some peptides induce apoptosis and reverse drug resistance by regulating these apoptotic pathways.

### 2.1 Peptides regulating apoptotic genes

Anti-apoptosis is the main mechanism of drug resistance in tumor cells (Igney and Krammer, 2002), and the production of anti-apoptosis is closely related to the imbalance of apoptotic genes. Apoptotic genes are mainly divided into two categories: genes promoting apoptosis and genes inhibiting apoptosis according to their functions. There are many kinds of Apoptotic-related genes, such as the Bcl-2 family, p53, Fas, APO-1, and caspase protein family. The main cause of drug resistance is to promote the downregulation of apoptotic genes such as p53 gene expression and inhibit the upregulation of apoptotic genes such as Bcl-2 gene expression. So regulating apoptotic genes to promote cancer cell apoptosis is to enhance its sensitivity to chemotherapy drugs (Wang H. et al., 2014), a feasible way to reverse drug resistance.

**FIGURE 1:**
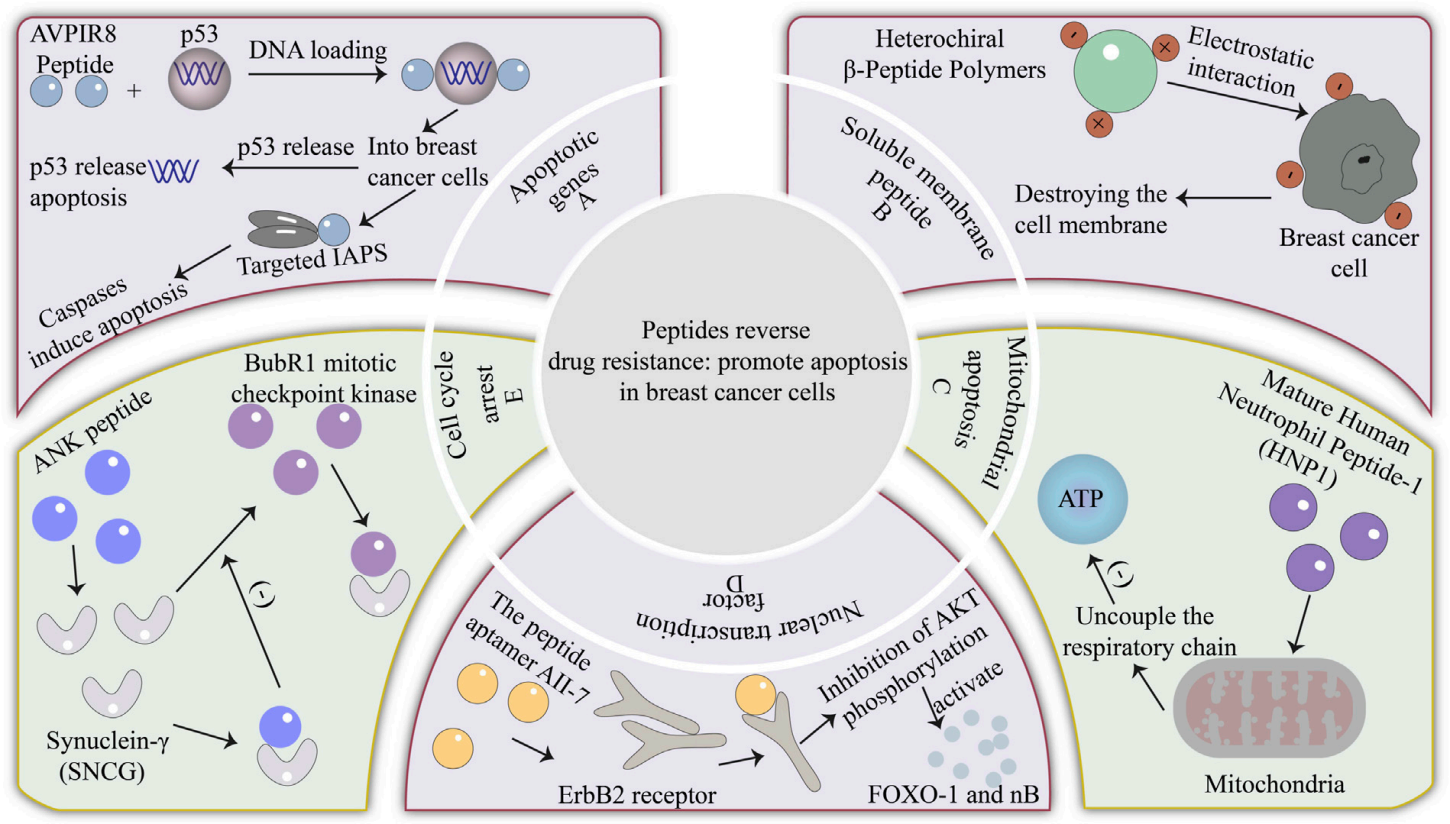
The mechanism of reversing drug resistance by promoting apoptosis of cancer cells. **(A)** Smac peptides are apoptosis peptides, the most widely studied is the Smac N-terminal tetrapeptide (AVPI), which has a strong binding force with the chain IAP, and the AVPI peptide and p53 DNA are co-delivered to induce apoptosis. **(B)** The synthesized amphiphilic β-peptide polymer is an ACP, which is composed of cationic and anionic residues. It can bind to the negative charge on the surface of cancer cells through electrostatic interaction, and then the hydrophobic part interacts with the phospholipid bilayer to destroy the cell membrane. **(C)** Mature human neutrophil peptide-1 first mediates the collapse of mitochondrial transmembrane potential in cells, decoupling the respiratory chain from oxidative phosphorylation, damaging the energy source of tumor cells, and leading to apoptosis of cancer cells. **(D)** The peptide aptamer All-7 can specifically bind to the ErbB2 receptor, interfere with the activation of the AKT pathway so it cannot regulate the nuclear transcription factor κB and FOXO-1 through the phosphorylation of AKT, and restore the sensitivity of tumor cells to paclitaxel. **(E)** As an inhibitor of the mechanism of action of SNCG protein, ANK peptide can bind to Synuclein-γ (SNCG) protein, prevent the interaction between SNCG protein and BubR1 mitotic checkpoint kinase, and the role of the mitotic checkpoint will return to normal.

The efficacy of paclitaxel, one of the first-line chemotherapy drugs for breast cancer, is limited because of the upregulation of the anti-apoptotic gene Bcl-2 (Kutuk and Letai, 2008). The corresponding countermeasure is to use some Bcl-2 inhibitors, such as navitoclax (ABT-263), but the effect of this method is limited. Therefore, the use of peptides such as NuBCP-9 peptide to combine Bcl-2 protein exposed Bcl-2 BH3 domain, making the Bcl-2 protein conformational changes, bax inhibition (Gupta et al., 2018), hinders the Bcl-2 gene production. In addition to the Bcl-2 gene plays an important role in tumor drug resistance, the decrease of p53 gene expression is another important reason for drug resistance. P53 mainly releases Smac by enhancing mitochondria (Yang et al., 2006; Carter et al., 2010), and Smac peptides are apoptotic peptides. The most widely studied is Smac N-terminal tetrapeptide (AVPI) (Wang, 2011), which has a strong binding force with the linked IAP. Therefore, AVPI peptide and p53 DNA can be synergistically delivered Figure 1. Not only increased the expression of p53 genes but also p53 can promote the release of Smac into the cytoplasm and induce apoptosis.

Caspase-dependent apoptosis also plays an important role in tumor drug resistance. Cells can activate caspase apoptotic genes with the participation of cytochrome C to cause cell death, but heat shock protein (HSP) inhibits the role of caspase apoptotic genes. For example, HSP70 makes breast cancer insensitive to cisplatin through this mechanism. Because HSP70 is expressed in both tumor cells and normal cells, many existing HSP70 inhibitors lack specificity and often lead to side effects (Leu et al., 2017). Therefore, a specific anti-HSP70 peptide was further developed. The anti-HSP70 peptide binds the HSP70 protein to prevent the inhibition of HSP70 on caspase and promote the apoptosis of cancer cells.

Therefore, the use of peptides to regulate apoptotic genes to induce breast cancer cell death is a new means of anti-drug resistance (Brown and Attardi, 2005). Most of these peptides upregulate the expression of the P53 gene or block the expression of the Bcl-2 gene, and enhance the production of apoptotic proteins such as caspase-3 to cope with the escape of cancer cells from apoptosis.

### 2.2 Soluble membrane peptides

As the name suggests, lysosomal peptides destroy the cell membrane. As a part of the innate immune defense barrier, the cell membrane will not be able to maintain cell survival after being destroyed, and the cells will also undergo apoptosis. It has been actively studied for the treatment of antibacterial, antiviral, and even anticancer (Guha et al., 2019). In anti-cancer treatment, a major advantage of lysosomal peptides is that they also have strong cytotoxicity to drug-resistant cancer cells (McPhee and Hancock, 2005), and can resist the resistance of tumor cells to chemotherapeutic drugs. However, at the same time, lysosomal peptides have certain limitations for anticancer therapy. First, the targeting effect of lysosomal peptides on tumor cells is not strong, and it is not able to accurately identify the subtle differences between tumor cells and normal cells (Leite et al., 2015; Kim et al., 2021). Secondly, the lysosomal peptide is not stable, and there are still difficulties in the way of administration. These are the reasons lysosomal peptides are not yet widely used in cancer treatment.

To kill tumor cells in a non-toxic manner, the first problem to be solved is the targeting of lysosomal peptides. Studies have designed a new type of lysosomal anticancer peptide (ACP) (Chen C. H. et al., 2022). The peptide changes the charge distribution of the peptide by purposefully introducing anionic residues (Andreev et al., 2010; Wiedman et al., 2017; Westerfield et al., 2019), then it can target the anionic lipids on the surface of cancer cells (Gaspar et al., 2012), thereby avoiding affecting healthy cells. The synthesized amphiphilic *β*-peptide polymer is also an ACP, which is composed of cationic and anionic residues Figure 1. It can bind to the negative charge on the surface of cancer cells through electrostatic interaction, and then the hydrophobic part interacts with the phospholipid bilayer, destroying the cell membrane and effectively resisting multiple drug resistance (Shao et al., 2022). Moreover, the novel membrane-soluble anticancer peptide is a neutral peptide that better targets cancer cells with subtle changes in cell surface pH. In addition to artificially designed and synthesized lysosomal peptides, natural lysosomal peptides such as HNP1-4, expressed primarily by neutrophils (Agerberth et al., 2000), can be found to enhance the plasma membrane permeability of tumor cells (Xu et al., 2008), selectively cytotoxic to cancer cells (Lichtenstein et al., 1986), and equally cytotoxic to drug-resistant cancer cells (Starr and Wimley, 2017).

In the face of the problem of lysing peptide administration route, nano-preparations are now used to assist the intravenous delivery of lysing peptides (Kong et al., 2021), reducing the hemolytic activity of lysing peptides and enhancing the stability of lysing peptides in plasma (Pino-Angeles and Lazaridis, 2018). Moreover, computer modeling is now combined with the research and development of lysosomal peptides, which promotes the speed of programming and design of new lysosomal peptides (Ulmschneider and Ulmschneider, 2018). It is believed that shortly, lysosomal peptides will be increasingly used in the treatment of malignant tumors such as breast cancer to improve the resistance of cancer cells to chemotherapeutic drugs.

### 2.3 Peptides promoting mitochondrial apoptosis pathway

Targeting the mitochondrial pathway is now also a strategy to reverse tumor drug resistance by inducing changes in mitochondrial function and promoting apoptosis of tumor cells. As for the mechanism of mitochondrial function changes, there are mainly several categories: changes in transmembrane potential, production of reactive oxygen species ROS, and release of apoptotic factors (Zhao et al., 2018; Soukupova and Rudolf, 2019).

Reducing mitochondrial transmembrane potential can block energy supply. The normal transmembrane potential is the premise to maintain the normal function of cells and even mitochondria. The reduction of mitochondrial transmembrane potential can uncouple the respiratory chain (Li et al., 2014), inhibit the production of ATP, block the energy supply of tumor cells and lead to cell death. Mature human neutrophil peptide-1 (HNP1) can induce a decrease in mitochondrial transmembrane potential (Li et al., 2014). Because the outer membrane of tumor cells contains more acidic phospholipids, and the cytoskeleton system of tumor cells is defective, the extracellular matrix components also change, and HNP1 is more likely to act on tumor cells and less affect normal cells (Muller et al., 2002). Mature human neutrophil peptide-1 first mediates the collapse of mitochondrial transmembrane potential in cells, then the respiratory chain is uncoupled with oxidative phosphorylation, damaging the energy source of tumor cells (Xu et al., 2005), leading to apoptosis of cancer cells and achieving the purpose of destroying drug resistance Figure 1.

Mitochondria produces and releases a large amount of reactive oxygen species ROS, which can induce cell death including cancer cells. Targeting mitochondria by designing peptides can destroy the mitochondrial membrane, increase its permeability, and release ROS (Ahmed S. et al., 2021). After a large amount of ROS enters the cytoplasm of breast cancer, it will destroy the cell structure and function by seizing electrons, leading to the death of cancer cells. At the same time, ROS can also lead to oxidative damage of mitochondrial components. Damage to mitochondrial DNA will further destroy mitochondrial oxidative phosphorylation, increase mitochondrial membrane potential (MMP) and release promotes cytochrome c (CytoC), and ultimately promote cell death (Yuan et al., 2021).

The release of apoptotic factors in mitochondria induces an intrinsic apoptosis pathway. The inducing factors of the initiation of the mitochondrial apoptosis pathway mainly include direct caspase activator, indirect caspase activator, and caspase-independent cell death effector (Martinou et al., 2000). The common apoptotic factor cytochrome C released by mitochondria is a direct caspase activator. Studies have shown that HER-2 peptide can mediate the increase of mitochondrial permeability, then release cytochrome C, and activate the caspase apoptotic gene family through a series of events. As a result, the cells move toward the apoptotic process of self-digestion (Shi et al., 2018). In addition to cytochrome C, reverse drug resistance and promote the death of tumor cells can also start from other apoptotic factors, such as caspase activator Smac, which is derived from the second mitochondria, and apoptosis-inducing factor AIF (Zimmermann et al., 2001). The mechanism is to induce the activation of apoptotic genes by increasing the release of mitochondrial apoptotic factors, and then induce a series of cascade reactions to induce apoptosis of cancer cells and overcome the multidrug resistance of malignant tumors such as breast cancer to many chemotherapeutic drugs such as doxorubicin.

### 2.4 Peptides regulating nuclear transcription factors

Studies have shown that nuclear transcription factors have a certain relationship with the generation of drug resistance in some malignant tumors. For example, the overexpression of homologous nuclear transcription factor Engrailed 1 (EN1) can lead to the resistance of basal-like breast cancer to the chemotherapeutic drug docetaxel (Sorolla et al., 2016). Some breast cancer cells activate the AKT pathway induced by the heterodimer of ErbB2. After AKT phosphorylation, the FOXO-1 transcription factor is inactivated, thereby inhibiting the expression of apoptotic proteins (Brunet et al., 1999). At the same time, AKT phosphorylation will also induce nuclear factor κB-mediated anti-apoptotic response and inhibit the expression of tumor suppressor (Romashkova and Makarov, 1999), resulting in paclitaxel resistance. Therefore, regulating the expression of nuclear transcription factors is also an idea to reverse drug resistance, including the use of peptides to control transcription factors.

Studies have used the peptide aptamer All-7, All-7 can specifically bind to the ErbB2 receptor, interfere with the activation of the AKT pathway Figure 1, and it cannot regulate the nuclear transcription factor κB (κB was first found in B lymphocytes because it binds to the B site of the immunoglobulin κ light chain gene enhancer and regulates the transcription of the immunoglobulin κ light chain, it is named nuclear transcription factor κB) and FOXO-1 through the phosphorylation of AKT, and restore the sensitivity of tumor cells to paclitaxel (Kunz et al., 2006). For the problem that some nuclear transcription factors are highly expressed in cancer cells, nuclear transcription factor interference peptides are a good solution. Because normal cells hardly express these nuclear transcription factors, they are less affected by nuclear transcription factor interference peptides. For example, the interference peptide (EN1-iPep) designed to block EN1 mainly inhibits the expression of EN1 by binding to the nuclear transcription factor EN1 through a dominant negative-like mechanism (Sorolla et al., 2016). Reduced expression of EN1 induces caspase-3-dependent apoptosis (Beltran et al., 2014), which can reverse the drug resistance of basal-like breast cancer cells. In addition, there is a dual-target hybrid peptide T10-ERK, which mainly interferes with the binding of MEK and ERK, hinders the activation of ERK (Sheng et al., 2016), inhibits the expression of related nuclear transcription factors to regulate the apoptosis of cancer cells, and achieves the purpose of promoting cancer cell death and overcoming drug resistance. In conclusion, it is a feasible scheme to improve the resistance of breast cancer cells to chemotherapeutic drugs by controlling the expression of nuclear transcription factors.

### 2.5 Cell cycle arrest peptides

Preventing tumor cell mitosis to arrest it in the division phase, that is, blocking the cell cycle is the mechanism of some chemotherapeutic drugs in the treatment of tumors. The commonly used anti-microtubule drugs such as paclitaxel and nocodazole block the cell cycle by causing spindle damage (Blajeski et al., 2002). At the same time based on this mechanism, it also produced resistance. Drug-resistant breast cancer cells can control or inhibit the expression of genes that hinder cell division by avoiding the examination of mitotic checkpoints, thereby successfully avoiding the results of mitotic arrest after chemotherapy.

Now some peptides can be successfully reversed inhibited cell cycle arrest, with the NT21MP peptide can enhance the expression of microRNA-335, and then through the Wnt/β-catenin signaling pathway to inhibit the expression of SETD8 target genes, so cells stagnate in G0/G1 phase. The ANK peptide, as an inhibitor of the mechanism of action of the Synuclein-γ (SNCG), can bind to the SNCG Figure 1, preventing the interaction of the SNCG with the BubR1 mitotic checkpoint kinase, and the role of the mitotic checkpoint will return to normal (Singh et al., 2007). Studies have also found that ALT peptides can also promote the cell cycle arrest of cancer cells. ALT peptides mainly block the tyrosine phosphorylation of p27, and the cyclin D-CDK4 dimer cannot be associated with p27Kip1 (Blain, 2008), thereby inhibiting CDK4 and CDK2. The cell cycle is blocked in the G1 phase, and ALT peptides can also prevent it from re-entering the cell cycle, which can enhance the effect of paclitaxel on cell cycle CDK4/6. After these peptides inhibit the mitosis of cancer cells and block the cell cycle, the body detects these arrested cells, which will induce them to gradually apoptosis and restore the toxicity of chemotherapeutic drugs such as paclitaxel to tumor cells.

## 3 Peptides reverse drug resistance: promote non-apoptotic cell death in breast cancer

In addition to reversing drug resistance through the apoptotic pathway, peptides can also be used to induce non-apoptotic regulatory cell death leading to tumor cell death (Yu et al., 2022), including autotropic cell death, and necroptosis. Here we mainly introduce two peptides that induce the lysosomal peptide apoptosis pathway and RIPK1-dependent necroptosis, respectively.

The lysosomal apoptosis pathway refers to the release of hydrolases in lysosomes to promote cell death, which is an autophagic cell death that can be applied to kill cancer cells and reverse the resistance of tumor cells to chemotherapeutic drugs. To induce the lysosomal apoptosis pathway, it is necessary to make the cell lysosomal membrane unstable and release hydrolases. There are proteases in these hydrolases, such as the common lysosomal marker cathepsin D (Sheng et al., 2015). These proteases are released into the cytoplasm and activate apoptotic effectors, such as caspase-3, to achieve the purpose of cell death. Therefore, the lysosomal pathway to induce apoptosis must first destroy the organelle membrane of lysosomes.

**FIGURE 2:**
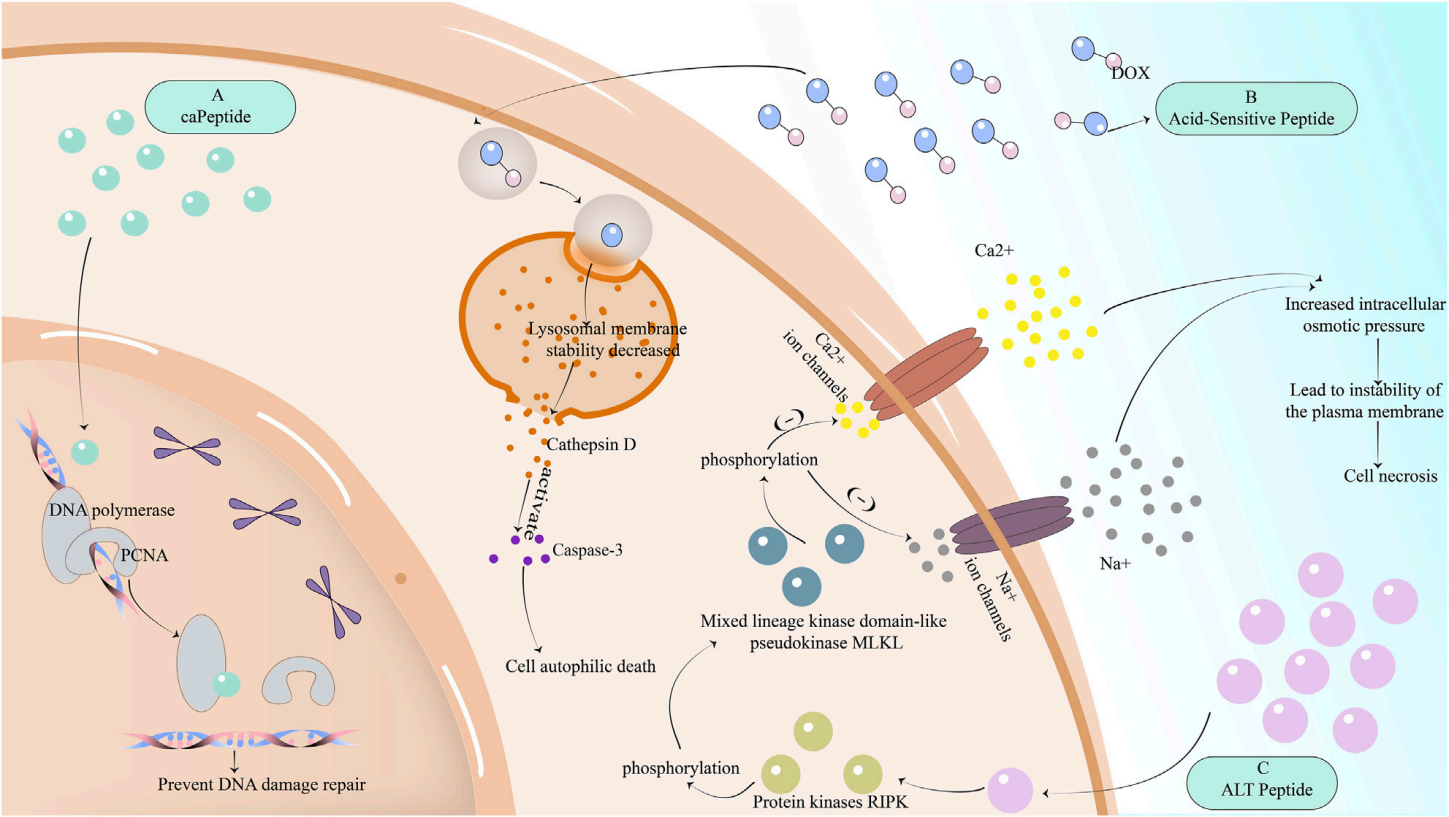
The mechanism of reversing drug resistance by partially inhibiting the DNA repair mechanism of cancer cells, or by promoting non-apoptotic regulatory cell death of cancer cells. **(A)** The peptide caPeptide that inhibits DNA repair in breast cancer cells can potentially bind to PCNA-interacting proteins such as POLD3 and increase genomic DNA damage. **(B)** Acid-sensitive peptides are internalized into lysosomes and other organelles through TfR-mediated endocytosis, resulting in decreased stability of lysosome membrane, enhanced permeability, the release of cathepsin D, activation of caspase-3, and induction of cell autophagic death. **(C)** ALT peptide first induces RIPK phosphorylation and then MLKL phosphorylation. MLKL phosphorylation will downregulate Ca2+ and Na + ion channels, resulting in increased intracellular osmotic pressure, instability of plasma membrane, and cell necrosis.

It has been proved that the accumulation of chloroquine in acidic vesicles can lead to the instability of endosomes and lysosomal membranes (Lubgan et al., 2009). Therefore, acid-sensitive peptides have been designed (Sheng et al., 2015), and acid-sensitive peptides are coupled with chemotherapy drugs. Acid-sensitive peptides can be internalized into lysosomes and other organelles through TfR-mediated endocytosis (Berczi et al., 1993), resulting in decreased lysosomal membrane stability, enhanced permeability, the release of cathepsin D, activation of caspase-3, and induction of cell autophagic death Figure 2.

The ALT peptide has been found to induce necrotic apoptosis of breast cancer cells through the mechanism of RIPK1 (Cekan et al., 2016; Jilishitz et al., 2021). The molecular mechanism of necroptosis is composed of protein kinases RIPK1 and RIPK3 and mixed lineage kinase domain-like pseudokinase MLKL. ALT peptide first induces RIPK phosphorylation and then MLKL phosphorylation. MLKL phosphorylation will downregulate Ca2+ and Na + ion channels, resulting in increased intracellular osmotic pressure, instability of plasma membrane, and cell necrosis Figure 2.

Non-apoptotic regulatory cell death such as cell autophagic death, and necroptosis has become a new method to induce tumor cell death, which is different from the apoptotic pathway and reverse drug resistance. With the deepening of research on non-apoptotic regulatory cell death, it will be more used in the treatment of cancer.

## 4 Peptides reverse drug resistance: inhibition of DNA repair mechanism in breast cancer cells

The mechanism of action represented by cisplatin in chemotherapeutic drugs for tumors is to damage the DNA of cancer cells, causing DNA damage response (DDR), which cannot be further replicated to cause cell death (Cekan et al., 2016). However, tumor cells later enhanced the repair of damaged DNA and developed resistance to chemotherapy drugs based on damaged DNA. Therefore, inhibiting DNA repair in breast cancer cells can reverse the drug resistance caused by the enhanced repair mechanism after DNA damage.

Ras-related nuclear protein (Ran-GTP) can mediate the repair of DNA damage, while Ran-RCC1 inhibitory peptide (RAN-IP) derived from Ran protein sequence Ran-RCC1 inhibits the formation of Ran-GTP (Haggag et al., 2017; Haggag et al., 2019) by competitively binding to RCC1 (Haggag et al., 2020), which not only reduces the level of Ran-GTP but also makes the nuclear input and output pathways regulated by Ran-GTP defective, inhibiting its role in enhancing DNA repair in cancer cells. Although RCC1 is also expressed in normal cells and can prevent the aging and death of normal cells due to DNA damage, the use of RAN-IP may have an impact on normal cells, but tumor cells are more affected after reducing Ran levels (Yuen et al., 2013; Yuen et al., 2016). On the other hand, proliferating cell nuclear antigen (PCNA) is also an important mechanism involved in DNA repair (Rassool and Tomkinson, 2010). PCNA can bind to POLD3, one of the dates subunits of DNA polymerase, causing DNA damage repair. To reverse drug resistance, another substance that can bind to POLD3 can be used to interfere with the role of PCNA. Scientists later found caPeptide, a peptide derived from caPCNA, which can potentially bind to PCNA-interacting proteins such as POLD3 (Lingeman et al., 2014), increase genomic DNA damage, and achieve the purpose of reversing the resistance of breast cancer to cisplatin-based chemotherapy drugs Figure 2.

Chemotherapy drugs with the mechanism of damaging cancer cell DNA, such as doxorubicin, mainly inhibit the effect of topoisomerase II (Mozaffari et al., 2021), but after the tumor cells strengthen the repair of DNA, the survival rate of these drugs is increased (Haggag et al., 2020). These peptides make the DNA damage of cancer cells irreparable by inhibiting the expression of Ran-GTP or PCNA. After the body detects DNA damage, the replication will be terminated, and the cells will then be cleared, successfully removing the resistance of cancer cells to doxorubicin and cisplatin.

## 5 Peptides reverse drug resistance: regulating the tumor microenvironment of breast cancer

Tumor microenvironment (TME) refers to the space around the tumor, which is composed of immune cells, matrix, and vascular system (Vasan et al., 2019). The tumor matrix is composed of non-malignant cell types such as cancer-associated fibroblasts (CAFs) and tumor mesenchymal stromal cells (MSCs) (Sahai et al., 2020). The tumor microenvironment often interferes with the immune clearance of tumor cells by producing some substances, promotes the growth of cancer cells, hinders the absorption of anticancer drugs, and promotes the progress of cancer. It is also the focus of cancer drug resistance research.

Cancer-associated fibroblasts (CAFs) can affect tumor cells through a variety of roles, such as leukocyte recruitment, immunosuppression, tumor microvascular formation, and extracellular matrix remodeling (Gascard and Tlsty, 2016). Due to the strong heterogeneity of CAFs, there has been a lack of therapy targeting CAFs. However, studies have found that many CAFs are related to adipose stromal cells (ASC) (Kolonin and DiGiovanni, 2019; Laurent et al., 2019). Therefore, a pro-apoptotic peptide D-CAN targeting ASC was found. D-CAN peptide indirectly inhibits the formation of CAFs by promoting ASC apoptosis and hindering the formation of CAFs by ASC and reverses the resistance of breast cancer to cisplatin and paclitaxel caused by ASC (Su et al., 2020).

**FIGURE 3:**
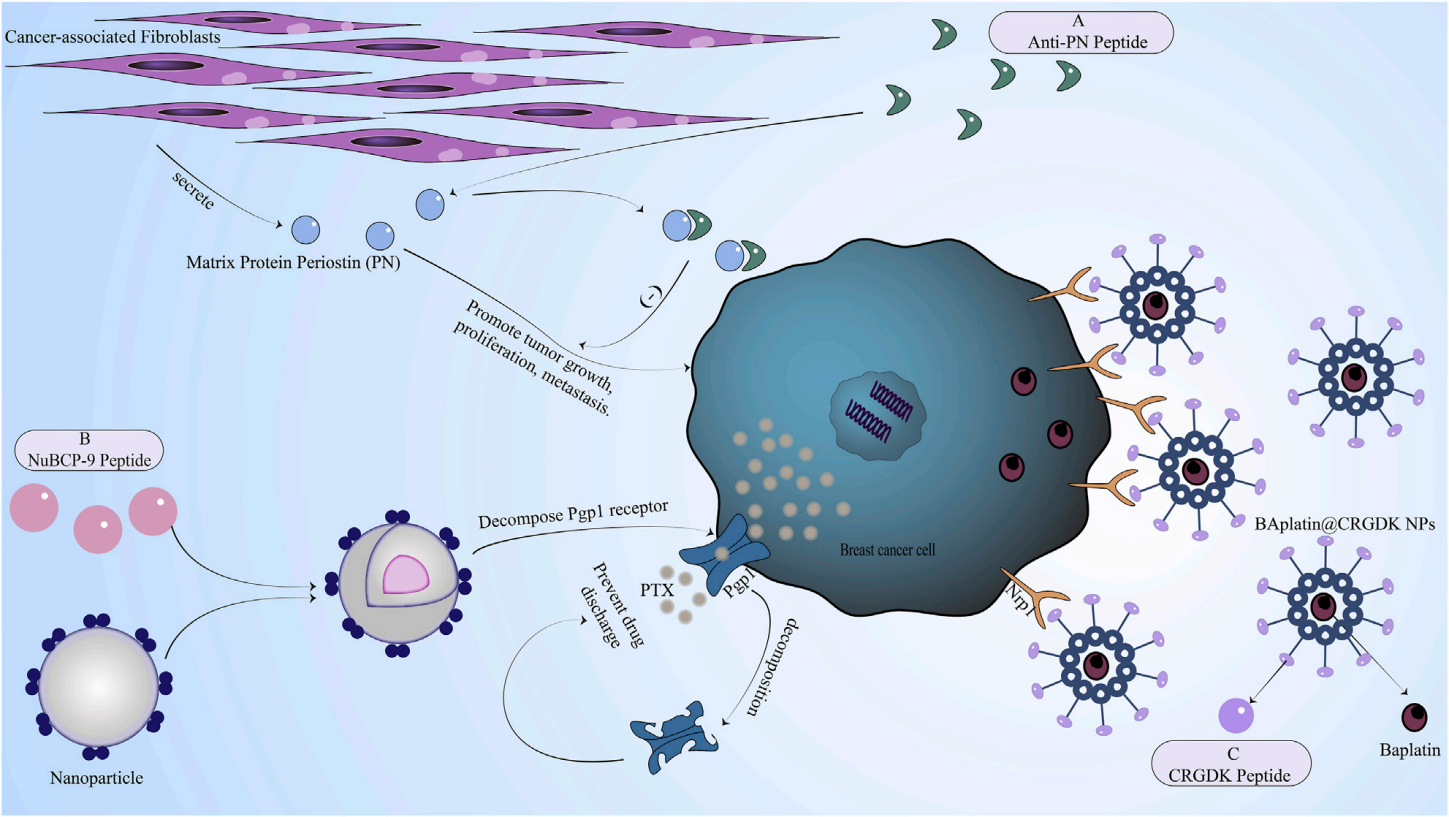
The mechanism of reversing drug resistance by regulating the tumor microenvironment of breast cancer, inhibiting chemotherapeutic drug efflux proteins in breast cancer cells, or enhancing breast cancer cell uptake of chemotherapy drugs. **(A)** Anti-PN peptide has a good affinity with PN secreted by cancer-associated fibroblasts in the tumor microenvironment. After binding to PN, the anti-PN peptide can inhibit the proliferation and metastasis of cancer cells induced by PN. **(B)** NuBCP-9 peptide increased drug concentration by inhibiting drug efflux mediated by pg efflux protein. **(C)** The CRGDK peptide binds to the overexpressed Nrp1 receptor of breast cancer cells through the CendR sequence to promote drug absorption.

In addition, a matrix protein periostin (PN) has been found, which is secreted by cancer-associated fibroblasts in the tumor microenvironment and can promote tumor growth, proliferation, metastasis, and angiogenesis through various mechanisms (Gonzalez-Gonzalez and Alonso, 2018; Liu et al., 2019). Moreover, PN is also related to the tolerance of a series of chemotherapeutic drugs such as doxorubicin, methotrexate, paclitaxel, cisplatin, and anti-angiogenesis therapy in breast cancer. The mechanism is mainly to enhance cell activity through the P13K/Akt signaling pathway. Because PN is mainly overexpressed in the tumor microenvironment of tumor cells such as breast cancer cells and is rarely expressed in normal cells. Therefore, inhibiting the production of PN is also a major strategy to reverse drug resistance. Researchers have screened a bioactive peptide anti-PN peptide from a phage library (Oo et al., 2021), which has a good affinity with PN by binding to integrin sites Figure 3. Anti-PN peptide binds to PN to inhibit the proliferation and metastasis of cancer cells induced by PN but has little effect on normal cells. Anti-PN peptide also reduces the activity of cancer cells by associating with AKT phosphorylation and surviving expression and reverses the resistance of breast cancer to chemotherapeutic drugs.

The tumor vascular system in the tumor microenvironment has a great influence on the growth of the tumor as a nutritional source of the tumor. Therefore, promoting the normalization of the tumor vascular system is also a method for treating cancer, especially cancer cells that are not sensitive to chemotherapy drugs (Dickson et al., 2007; Shen et al., 2012). For example, DEAE-glucan has an anti-angiogenesis effect by inducing β-interferon production (Bakrania et al., 2017). In addition, human neutrophil peptide-1 (HNP1) (Li et al., 2014), through the formation of a ternary complex with fibronectin and α5β1 integrin, further inhibits VEGF-induced endothelial cell proliferation, thereby making VEGF-mediated angiogenesis impossible, and ultimately promoting the normalization of tumor microvascular.

## 6 Peptides reverse drug resistance: inhibiting chemotherapeutic drug efflux proteins in breast cancer cells

The resistance of tumors to chemotherapeutic drugs, especially multidrug resistance, is closely related to the increased expression of efflux proteins in cancer cells. The common efflux proteins related to drug resistance are P-glycoprotein (pg) and multidrug resistance protein (MRP) (Sadava et al., 2002). Efflux proteins have a positive significance in normal cells, which can discharge toxic and harmful wastes from cells and ensure normal physiological functions of cells. However, the efflux protein in cancer cells can expel chemotherapeutic drugs from cells and reduce the content of drugs in cells (Seelig and Gatlik-Landwojtowicz, 2005), thereby reducing the biological efficacy and toxicity of anticancer drugs. Therefore, efflux proteins have also become a research direction for reversing drug resistance, including reducing the expression of efflux proteins in cancer cells or avoiding efflux proteins.

In breast cancer based on efflux protein reversal resistance strategy, some use drug delivery systems (Yang et al., 2008), then peptides and chemotherapy drugs such as doxorubicin, paclitaxel, and others couple into a common system, the system is to bypass the peptide efflux protein role. The most commonly used peptide in drug delivery systems is cell-penetrating peptide. For example, the small molecule synthetic peptide TAT is a cell-penetrating peptide (Mozaffari et al., 2021), which can successfully avoid efflux proteins such as P-glycoprotein (pg) and can significantly increase the drug concentration in cancer cells. Of course, the use of peptides and the recognition of efflux proteins on the drug surface can also increase the accumulation of intracellular drugs (Lindgren et al., 2006). Another important reason why the drug delivery system can avoid being excreted by the efflux protein is that the molecular weight of the drug delivery system is too large to exceed the size of the efflux protein and naturally cannot be excreted by the efflux protein. In addition, the use of T10-ERK peptide can also interfere with drug efflux mediated by efflux proteins such as P-glycoprotein (pg) to increase drug concentration (Sheng et al., 2016). There are also methods for using Pgp1 inhibitors, such as the inclusion of NuBCP-9 peptide in PLA-PEG-PPG-PEG nanoparticles Figure 3, which can decompose Pgp1 receptors in the acidic environment of the tumor to ensure that the drug is not expelled from the cell (Gupta et al., 2018).

In addition to the drug efflux mechanism related to efflux proteins, there are some other mechanisms. The common one is the overexpression of ATP-binding cassette (ABC) transporters in cancer cells. Therefore, the use of peptides to inhibit the expression of ABC transporters can be considered. ABC transporters cannot convert ATP-binding and hydrolyzed energy into mechanical energy (Dong et al., 2005), and their mediated drug transmembrane transport outflow will also be inhibited. Whether it is inhibiting efflux proteins or inhibiting transporters, it is to increase the drug concentration in cancer cells, enhance drug toxicity, and reverse drug resistance.

## 7 Peptides reverse drug resistance: enhance breast cancer cell uptake of chemotherapy drugs

A drug delivery system is a good method to enhance drug absorption. It is mainly composed of polymer materials, inorganic materials, stabilizers, accelerators (promoting drug dissolution and absorption), and blockers (controlling drug release). Nowadays, more and more peptides are used in drug delivery systems. Some of them play a role in surface modification to mediate targeted delivery and enhance endocytosis, while some peptides are only used as polymer carriers to deliver drugs (Ahmed K. S. et al., 2021). These effects are intended to enhance the absorption of drugs for tumor cells and can fight drug resistance, because drug absorption disorder is also one of the reasons for cancer cells are not sensitive to chemotherapy drugs.

### 7.1 Peptides modify chemotherapeutic drugs to target delivery to cancer cells

Peptides have the characteristics of small molecular weight, easy chemical synthesis, and antibodies. Based on these advantages, peptides can be used as a cancer-selective targeting agent (Falciani et al., 2009). Peptides are conjugated to the carrier of the drug delivery system, some for targeted delivery and others to mediate enhanced drug endocytosis. The first is targeted delivery, which is based on the overexpression of tumor cells or specific substances. With these substances as receptors, peptides in the drug delivery system can specifically bind these receptors to achieve targeted drug delivery. In breast cancer cells, neuropilin-1 receptor (Nrp1) (Bai et al., 2019), membrane protein receptor-related protein (LRP) (Depau et al., 2017), GRPR (Wang et al., 2016), human epidermal growth factor receptor 2 (HER-2) (Shi et al., 2018), VEGFR1 (Aldughaim et al., 2020), etc. Are frequently overexpressed.

Many peptides have been found to specifically bind these overexpressed receptors in breast cancer cells. For example, CRGDK peptide (CysArg-Gly-Asp-Lys) can bind to Nrp1 through the CendR sequence (Bai et al., 2019) Figure 3, while cancer-selective tetrapeptide (NT4) can bind to membrane protein receptor-related protein (LRP) and heparan sulfate chain on its glycan (Depau et al., 2017). NT4 peptide can also inhibit the adsorption and migration of matrix proteins and affect cell movement after binding to these specific targets (Brunetti et al., 2016). Bombesin (Bn) isolated from amphibian tissues can specifically bind to GRPR receptors and has been used in experiments to deliver doxorubicin, successfully reversing drug resistance (Wang et al., 2016). HER-2 peptide targeting the HER-2 receptor is an analog of trastuzumab and a good surface modifier for a common drug delivery system targeting the HER-2 receptor (Shi et al., 2018). There is also a p700 peptide, which is an amino acid fragment at the C-terminus of TIMP3 and can specifically bind to receptors such as VEGFR1 and FGFR1-4 (Soudy et al., 2013). These peptides as the surface modification of the drug delivery system can be well-targeted delivery of doxorubicin, cisplatin, and other chemotherapy drugs, thereby more drugs into tumor cells.

There is also a method to enhance the release of chemotherapeutic drugs, because cathepsin B is overexpressed in cancer cells, and cathepsin B can catalyze the hydrolysis of GFLG peptide (Zhi et al., 2019). Therefore, the modification of GFLG on the drug delivery system can achieve the targeted release of chemotherapeutic drugs.

Enhancing endocytosis is also a means of increasing drug concentration in cancer cells. Among them, the transferrin receptor (TfR) -mediated endocytosis pathway has attracted much attention (Sheng et al., 2016). Tf is conjugated to the drug delivery system, and the combination of Tf and TfR can increase the number of chemotherapeutic drugs entering tumor cells (Sheng et al., 2015). The method of enhancing endocytosis also uses cell-penetrating peptides, which can carry macromolecules through the cell membrane. For example, the new cell-penetrating peptide YTA2 successfully solved the problem of MTX lack of transporters during transport (Rousselle et al., 2000), and MTX was delivered to tumor cells (Lindgren et al., 2006). And adjusting the hydrophilic side and hydrophobic side of the peptide affixed to the surface of the drug delivery system to facilitate the endocytosis of the chemotherapy drug into the cancer cell (Bai et al., 2019).

### 7.2 Peptides are only used as carriers to deliver chemotherapeutic drugs

Polymer materials, an important part of drug delivery systems, can be divided into natural polymer materials, semi-synthetic polymer materials, and synthetic polymer materials. Nanocarriers are very common in drug delivery systems. Commonly used nanocarriers include liposomes, dendritic polymers, and metal nanoparticles (Markman et al., 2013). Nanocarriers can encapsulate the drug well and release the drug slowly, ensuring the local concentration of the drug and prolonging the residence time of the drug in the cell.

Peptides can be used as a natural polymer material carrier to encapsulate drugs. Although peptides are limited by proteolytic enzymes in drug delivery systems, many studies have ensured peptide stability by substituting for unstable sites in enzymatic reactions, such as a protein-stabilized decapeptide WxEYAAQrFL. In addition, a cyclic peptide nanotube made of cyclic octapeptide to load doxorubicin not only has high encapsulation efficiency but also has good dispersion (Wang H. et al., 2014), which can increase the uptake of doxorubicin by cancer cells. There is also the use of binding peptide dendrimers as carriers to deliver drugs because their good biocompatibility can enhance the permeability of drugs (Zhu et al., 2021). The molecular weight of AmPD KK2 peptide in the synthesized peptide dendrites is smaller than that of AmPD KK2K4, and the balance between hydrophobicity and hydrophilicity of AmPD KK2 peptide is better maintained, so it can be better taken up by cells. In summary, peptides have great potential as a natural polymer material that can encapsulate chemotherapy drugs.

## 8 Peptides as a drug moiety: current challenges and possible solutions

As an anticancer peptide with the effect of reversing the resistance of chemotherapeutic drugs and treating malignant tumors such as breast cancer, it has the characteristics of easy synthesis and modification, various modes of administration, and difficulty in producing multidrug resistance (Chen G. et al., 2022). Moreover, the peptide itself has strong targeting, small adverse reactions, and easy separation and transformation (Qin et al., 2022). Although many peptides have been found to have good anti-tumor activity, the development of peptide drugs faces many challenges. For example, the yield of natural peptides is low, and the later artificial synthesis is expensive (Akbarian and Chen, 2022); at the same time, the peptide will produce immunogenicity in the host, and the presence of peptidase in the serum makes the half-life of the peptide shorter (Duran-Lobato et al., 2021). Secondly, *in vivo*, digestive enzymes will decompose peptides (Muttenthaler et al., 2021). In addition, the anti-tumor mechanism of peptide compounds is not fully understood, and the related clinical and pharmacological studies are still less, which still needs further study.

At present, the targeting, stability, low toxicity, solubility, and half-life of peptides can be improved by using different types of modification, such as peptide engineering, new preparations, and peptide coupling (Mitra et al., 2020). It has been found that many peptide drugs have entered clinical trials or have been approved for marketing (Anand et al., 2023). These peptides can be used as peptide hormones, radionuclide carriers, peptide vaccines, cytotoxic drug carriers, and anticancer drugs for cancer treatment in different ways (Gu et al., 2021). For example, gonadotropin-releasing hormone receptor (LHRH) agonists such as leuprolide acetate for the treatment of prostate cancer inhibit the proliferation of prostate cancer cells by down-regulating the expression of LHRH in the pituitary, inhibiting the release of follicle-stimulating hormone (FSH) and reducing the production of testosterone (Luo et al., 2023). Octreotide is a radionuclide carrier. After radioactive labeling with indium 111, it can be attached to tumor cells with somatostatin receptors, and the location of tumor cells can be determined by detecting radioactive octreotide (Hijazi, 2021). The drug carrier AEZS-108 exerts its anticancer effect by directly targeting cancer cells expressing luteinizing hormone-releasing hormone receptors by coupling the peptide with the chemotherapeutic drug doxorubicin (Yang et al., 2023). Although peptide drugs have good clinical value in cancer treatment, it is still necessary to overcome the limitations of peptides. It is of great significance to further discover new anticancer peptides or overcome their shortcomings by a modification to reduce cancer mortality.

## 9 Conclusion and future prospect

With the increasing incidence of breast cancer in recent years (Liu et al., 2022), the problem of breast cancer resistance to chemotherapeutic drugs has also received more attention. Drug resistance has become a huge obstacle to the effective treatment of breast cancer with chemotherapeutic drugs (Wang H. et al., 2014). First-line anticancer drugs with good efficacy, such as doxorubicin, paclitaxel, and are difficult to control tumor growth and metastasis, which shortens the survival of breast cancer patients. There are many reasons for drug resistance, including defects in cancer cell apoptosis, enhanced DNA repair function of tumor cells, reduced drug uptake, enhanced efflux mechanisms, and changes in targets (Bai et al., 2019). To reverse drug resistance, it is necessary to start with the mechanism of these drug resistances. Many studies are exploring ways to solve the problem of drug resistance, and the use of peptides to overcome drug resistance is a feasible solution. Here, we summarize the strategies of different peptides for reversing breast cancer drug resistance and elaborate on the mechanism of these peptides.

We divided the mechanism of action of the peptides currently studied on reversing breast cancer resistance into six categories, including promoting cancer cell apoptosis, promoting non-apoptotic regulatory cell death of cancer cells, inhibiting DNA repair mechanisms of cancer cells, improving tumor microenvironment, inhibiting drug efflux mechanisms and enhancing drug uptake. Among them, promoting apoptosis is divided into several mechanisms, including regulating apoptotic genes (up-regulating pro-apoptotic genes and down-regulating anti-apoptotic genes), using lysosomal peptides, inducing mitochondrial apoptotic pathways, regulating nuclear transcription factors, and arresting cell cycle. Promoting non-apoptotic regulatory cell death includes promoting RIPK1-dependent necrosis and inducing lysosomal-dependent cell death. Finally, the enhancement of drug uptake can be achieved by peptide modified drug delivery system to achieve targeted drug delivery, enhance endocytosis and enhance drug release, or the peptide is only used as a carrier to improve the encapsulation efficiency and dispersion of the drug and increase the drug uptake.

Most of the peptides mentioned in this article are chemically synthesized, and only a few are derived from animals and plants. However, studies have shown that some natural peptides, especially marine peptides, have anti-drug-resistant tumors and can significantly reduce the activity of drug-resistant tumor cells (Ahmed S. et al., 2021) Table 2. However, whether these natural peptides can specifically target breast cancer and reverse its chemotherapy resistance needs further study. Because the peptide molecular weight is small and easy to synthesize (Falciani et al., 2009), it has great advantages in the study of reversing breast cancer resistance. Many peptides that effectively reverse breast cancer resistance have been explored, such as The 8-mer peptide derived from β-fetoprotein inhibits the growth of tamoxifen-resistant estrogen receptor-positive breast cancer cells (Bennett et al., 2002), and Membrane-Lytic Peptide (Chen C. H. et al., 2022), Acid-Sensitive Peptide (Sheng et al., 2015), RanGTP inhibitory peptide (RAN-IP) (Haggag et al., 2020). We hope to use these peptides to overcome the resistance of breast cancer to chemotherapeutic drugs, enhance the therapeutic effect of anticancer drugs, improve the survival rate of breast cancer patients, especially metastatic breast cancer patients, and prolong the life of patients. Although these peptides have shown the effectiveness of overcoming breast cancer resistance in experiments, we need to pay attention to the fact that many experiments are still *in vitro* experiments, and almost no *in vivo* experiments are carried out. Therefore, there may be a long way to go to truly apply them in clinical practice.

**TABLE 2:** Natural peptides reversing chemotherapy resistance and its mechanism.

Peptide name	Source	Active derivative	Reversed drugs	Reversing resistance mechanisms	References
Hemiasterlin and Hemiasterlin C	Sponge (*Hemiasterella minor, Auletta* sp.*, Cymbastela* sp.*,* and *Siphonochalina* sp.)	Linear tripeptide		Antiproliferative effect; Microtubules Depolymerization; G2/M phase arrest	Gamble et al. (1999)
HTI-286	Sponge (*Hemiasterella minor, Auletta* sp.*, Cymbastela* sp.*,* and *Siphonochalina* sp.)		Paclitaxel, vincristine, vinblastine, colchicine, and doxorubicin Docetaxel and vinorelbine	Antiproliferative effect	Loganzo et al. (2003)
Milnamide A-D	*Auletta* sp.			Microtubules depolymerization	Sonnenschein et al. (2004)
Symplostatin 1	cyanobacteria of the genus Symploca	Linear Pentapeptide	Taxol and vinblastine	Bcl2 ↓; Microtubules depolymerization	Mooberry et al. (2003)
Cryptophycin	*Nostoc* sp.	Cyclic depsipeptide	paclitaxel and colcemid	Microtubules Depolymerization; P-gp↓	Smith et al. (1994)
Geodiamolide D-E	Sponge (*Auletta* sp. And *Geodia corticostylifera*)			Actin filament disruption	Sonnenschein et al. (2004)
Kulokekahilide-2	Mollusk (Philinopsis speciosa)			↓ cell viability	Nakao et al. (2004)
Stylopeptide 2	Sponge (*Stylotella* sp.)	Cyclic decapeptide		Antiproliferative effect	Brennan et al. (2008)
Ohmyungsamycin A-B	Bacteria (*Streptomyces strain SNJ042*)	Cyclic depsipeptides		↓ cell viability	Um et al. (2013)
Largazole	Cyanobacteria (*Symploca* sp.)	Cyclic depsipeptide		↓ cancer cell growth	Taori et al. (2008)
C-phycocyanin	Cyanobacteria (*Limnothrix* sp. *NS01* and *Spirulina platensis*)	Peptide		DNA fragmentation; Caspase-9 ↑; cyt c ↑; Bcl2↓; Bax↑; PARP ↑; Stat3 ↓ G1 - G2 phase arrest (cyclin D1↓, cyclin E↓; p21↑) Antiangiogenic (VEGFR2 ↓ and MMP- 9 ↓) AKT inhibition; γ-H2AX ↑; Production of ROS and singlet oxygen radicals	Jiang et al. (2018)
Pipecolidepsin A-B	Sponge (*Homophymia lamellosa*)			↓ cell viability	Coello et al. (2014)
Pembamide	Sponge (*Cribrochalina* sp.)	N-methylated linear peptide		↓ cell viability	Coello et al. (2014)
Callyptide A	Sponge (*Callyspongia* sp.)	Cyclic peptide		↓ cell viability	Shaala et al. (2016)
DZ-2384	Ascidia (*Diazona angulate*)	Macrocyclic peptide	Taxane and vinca alkaloid	Microtubule depolymerization	Wieczorek et al. (2016)
Hymenochirin-1B	*Hymenochirus boettgeri* (Pipidae)			↓ cell viability	Attoub et al. (2013)
Alyteserin-2a	the midwife toad *Alytes obstetricans*			Antiproliferative Effect; TGF-β↓	Conlon et al. (2013)
Micro cionamides A-B	the Philippine marine sponge *Clathria (Thalysias) abietina*			↓ cell viability	Davis et al. (2004)
Kahalalide F	Mollusk (*Elysia rufescens*)	Cyclic depsipeptide		PI3K-AKT inhibition; ErbB3 depletion	Suarez et al. (2003)
Elisidepsin	Mollusk (*Elysia rufescens*)		oxaliplatin, cisplatin, 5-FU, gemcitabine and lapatinib	↑ Ca2+ influx; perturbations of membrane conductivity; the formation of giant membranous vesicles; ErbB3 depletion; Bcl2 ↓	Serova et al. (2013)

## Figures and Tables

**Table:** 

**HER2**	Human epidermal growth factor receptor 2
**ER**	Estrogen receptor
**PR**	Progesterone receptor
**TNBC**	Triple-negative breast cancer
**DOX**	Doxorubicin
**MTX**	Methotrexate
**CDDP**	Cisplatin
**PTX**	Paclitaxel
**CPP**	Cell penetrating peptide
**BCP**	Bioactive cationic peptide
**ABT-263**	Navitoclax
**AVPI**	Smac N-terminal tetrapeptide
**HSP**	Heat shock protein
**ACP**	lysosomal anticancer peptide
**HNP1**	Human neutrophil peptide-1
**MMP**	Mitochondrial membrane potential
**CytoC**	Promotes cytochrome c
**EN1**	Homologous nuclear transcription factor Engrailed 1
**EN1-iPep**	EN1 interference peptide
**SNCG**	Synuclein-γ
**DDR**	DNA damage response
**Ran-GTP**	Ras-related nuclear protein
**RAN-IP**	Ran-RCC1 inhibitory peptide
**PCNA**	Proliferating cell nuclear antigen
**TME**	Tumor microenvironment
**CAFs**	Cancer-associated fibroblasts
**MSCs**	Tumor mesenchymal stromal cells
**ASC**	Adipose stromal cells
**PN**	Periostin
**Pg**	P-glycoprotein
**MRP**	Multidrug resistance protein
**ABC**	ATP-binding cassette
**Nrp1**	Neuropilin-1 receptor
**LRP**	Membrane protein receptor-related protein
**NT4**	Cancer selective tetrapeptide
**GRPR**	Gastrin Releasing Peptide Receptor
**VEGFR1**	Vascular endothelial growth factor
**Bn**	Bombesin
**TfR**	Transferrin receptor
